# Report from the Medical Wards of the Illinois General Hospital

**Published:** 1853-10

**Authors:** N. S. Davis

**Affiliations:** One of the Physicians to the Hospital, and Prof. of Pathology, Practice of Medicine, and Clinical Medicine in Rush Medical College


### THE NORTH-WESTERN MEDICAL AND SURGICAL JOURNAL.

### NEW SERIES.

VOL. II. OCTOBER, 1853. No. 6.

### ORIGINAL COMMUNICATIONS.

ART. I.—*Report from the Medical Wards of the Illinois General Hospital*. By N. S. Davis, M.D., one of the Physicians to the Hospital, and Prof. of Pathology, Practice of Medicine, and Clinical Medicine in Rush Medical College.

(*Concluded from last Number.*)

Mr. H., aged 22 years, was admitted to the hospital June 28th, after having been sick little more than one week.

Present condition: cheeks deeply flushed and hot; skin generally dry and above the natural temperature; eyes injected and heavy; lips, mouth and tongue dry; the latter covered in the middle with a dark brown fur; pulse 100 per minute and easily compressed; breathing short and oppressed; mixture of dry and moist bronchial rales with dulness on percussion over the inferior and posterior portions of the lungs; abdomen moderately distended and tympanitic, with slight tenderness to pressure in the umbilical region; dark liquid evacuations from the bowels from three to six times a day for several days past; urine scanty and high colored; mind dull, with slight wandering at times. He was ordered beef tea well salted for nourishment, and the emulsion of oil of turpentine, (the formula already given) and laudanum in doses of one fluid drachm every three hours. The next morning he was found expectorating more freely, discharges from the bowels less frequent, and the frequency of the pulse and heat of skin diminished. Complained of feeling weak. Continued the same treatment with the addition of two grains of quinine to each dose of the emulsion. The third day the bowels were less tympanitic and the discharges less liquid, pulse 90 per minute and weak, tongue and mouth more moist, but breathing still short and oppressed. Ordered the emulsion and quinine continued every four hours with a teaspoonful of the following expectorant between each dose, viz.:

℞ Compound Honey of Squills and Senega, ℥j.
Tinct. Opii. et Camph. ℥jj. Mix.

On the morning of the fourth day there was an entire intermission in the general febrile symptoms, the skin, mouth, and tongue being moist, pulse only 85 per minute, though feeble, and expectoration free, presenting a muco-purulent character. Abdomen still slightly tympanitic, with two or three discharges during the last twenty-four hours. Ordered the emulsion continued every four hours with only two doses of quinine of 3 grs. each, given during the forenoon. Omit the expectorant, and allow in addition to the beef-tea, boiled milk and flour once or twice a day.

On the fifth day I learned that the patient had a slight return of fever during the previous afternoon, but is still further improved to-day. Directed the same treatment to be continued with the addition of an infusion of lycopus virginicus, given in doses of a wine glassful four times a day. This treatment was continued until the eighth day after admission, when the bowels had become regular, the respiration free, and the convalescence complete. Being much debilitated with some remaining expectoration, I ordered him to continue the lycopus virginicus in the form of infusion, and moderate doses of tinct. cinchonæ and tinct. valerian, equal parts, as a tonic. From this time he continued daily to improve in strength and appetite, and was discharged well the following week.

This was a well marked case with severe intestinal and moderate pulmonary complication. In the advanced stages of those cases where the lungs are more extensively involved, I have used, in addition to other remedies, an infusion of the lycopus virginicus with the rad. senega, with very good effects. On the other hand, when the cases have come into the hospital during the first week of their progress with marked pneumonic inflammation or congestion, I have used for twenty-four or forty-eight hours a powder composed of tartarized antimony, one-sixth of a grain, pulv. opii., one grain, and calomel two grs, every three or four hours, not only without injury, but with the best effects.

It is proper to remark, however, that the action of antimony and calomel must be very carefully guarded and, continued only for two or three days at longest, or they will induce a troublesome degree of intestinal irritation, requiring the prompt use of the emulsion of turpentine and laudanum to check.

In a few instances I have found a pill, composed of opium one grain and nitrate of silver half a grain, to control the intestinal affection, better than the emulsion. In other cases where the general typhoid symptoms were strongly marked in connection with intestinal disease, I have found the emulsion, the nit. argent., and opii., both to fail when fifteen or twenty grain doses of common salt, combined with from one to two grains of opium, and repeated every three or four hours, has promptly checked the progress of the disease and determined an early convalescence.

The reader will infer from the foregoing that I am neither an an advocate of simple *expectancy* in the treatment of continued fevers, nor a believer in the absolute necessity of having them run a specific course of from three to six weeks' duration.

It may be worthy of remark, that one of the cases recorded as typhus, when admitted into the hospital about the end of the first week of the disease, was universally covered with *sudamina* so copiously, that it caused a general desquamation of the cuticle. The same case also presented diffused inflammation of the pharynx and tonsils.

Of the eight cases of dysentery treated, a large majority were admitted in the advanced stage of the disease, when the general symptoms had put on a distinct typhoid character, and more or less purulent matter was mixed with blood in the discharges. In such cases the general course of treatment was very similar to that already detailed as applicable to the strongly intestinal cases of typhoid fever; to which was added the frequent use of *cold water* and laudanum in the form of enemas, and sometimes blisters over the abdomen.

In two cases the efficacy of pretty large doses of opium was tested. They were both admitted during the first week after the commencement of the disease, and the discharges were very frequent, consisting of mucous streaked with blood, and accompanied by much pain and fever, but not of a typhoid character. They were each ordered *three* grains of opium at bed-time. This dose produced entire suspension of the discharges and pain during the whole night; the patients also slept soundly and sweat freely, expressing themselves as feeling much better in the morning. Discharges of the same character as at first returned during the day, but without much pain. The same quantity of opium was given the second night and with precisely the same effects as before, but the discharges returning again the next morning, one of the patients was put upon a pill of nitrate of silver one-third of a grain, and opium one grain, every three hours, with a blister over the transverse arch of the colon which was tender to pressure, and the other patient was put on the emulsion of turpentine and laudanum with two grs. of quinine added to each morning dose, and both recovered. In a few cases presenting strongly marked typhoid symptoms, with discharges of bloody serum rather than mucous, I have found the chloride of sodium and opium most promptly beneficial in arresting the discharges and determining convalescence. In such cases I generally give fifteen grs. of common salt, with from one to two of opium at one dose, and repeat it every two, three or four hours, according to the frequency of the discharges.

One of the cases included under the head of dysentery, and which terminated fatally, is perhaps worthy of a more detailed notice:

Mr. C., aged about forty years, native of Ireland, was admitted into the hospital about the 20th of December, with very extensive chronic Eczema. He stated that he was attacked with the intermittent fever during the autumn previous, which continued several weeks, during which time the eruption made its appearance over the surface of the body. At the time of admission his pulse was 90 per minute and feeble, lips pale; bowels regular as to time, but evacuations liquid; some thirst, and digestion impaired. An eruption of eczemous vesicles existed over the entire cutaneous surface. From the knees to the ankles and across the lower part of the abdomen, including the groins, the skin presented a continuous red surface, from which oozed constantly a yellow serous fluid that by evaporation formed thin yellow scabs. Over the rest of the surface the eruption was more diffused, but everywhere it was accompanied by an intolerable itching. He was ordered the persesqui nitrate of iron in doses of eight drops three times a day, with a wash to apply to the skin twice a day, consisting of bichloride of mercury one grain, and water one pint. This treatment was continued until the 7th of January, with a very moderate degree of improvement. The patient complained, at times, of much burning or sense of heat in his stomach, and the evacuations from the bowels remained liquid. He was now ordered a pill composed of one-twentieth of a grain of arsenious acid with half a grain of opium each morning, noon, and night, and a wash consisting of a weak solution of chloride of lime. The next morning he was found with active diarrhœa, accompanied by an inordinate burning sensation throughout the alimentary canal, with redness and dryness of the mucous membrane of the mouth. The pills were omitted and the patient directed to take two grains of opium every three hours until the diarrhœa ceased.

Jan. 9th. The opium did not arrest the discharges which continue frequent, and this morning present a dysenteric character, being composed chiefly of mucous streaked with blood. Directed a powder composed of pulv. opii. one grain, and acetate of lead two grains, to be given every four hours, with a teaspoonful of the emulsion of oil turpentine and laudanum between each dose.

10th. Discharges continue the same as yesterday, and are accompanied by most severe *scalding* pain. Continue the same treatment; and direct an enema of cold water four oz. and tinct. opii. one drachm morning and evening.

11th. No improvement. Tongue and mouth very red; scalding pain in the abdomen very severe. Directed a pill composed of nitrate of silver one-fourth grain and ext. hyosciamus two grains every three hours; an enema of sulphate of iron twenty grains to the pint of cold water, and a blister over the epigastrium.

12th. Discharges still continue and accompanied by much prostration. The pulse feeble and frequent, with coldness of the extremities. Directed brandy punch alternated with carb. ammonia internally, and an enema of nitrate of silver in the proportion of five grs. to the oz. of water, but it produced no effect and the patient died the following morning.

*Remarks.*—Consent for a post-mortem examination could not be obtained; but from the appearance of the mucous membrane of the mouth and fauces, and the peculiar burning or scalding nature of the pain, apparently through the whole alimentary canal, I have no doubt but there was a direct extension of the cutaneous disease to the mucous membranes. I say *extension*, because there was no recession of the eruption from the surface. It continued its usual appearance externally up to the last day of life. That the intestinal disease was truly an extension of the cutaneous one, and not simple complication of dysentery, was further corroborated by the fact that the eczemous eruption extended from the face over the pro-labia into the mouth, and from the lower part of the abdomen to the verge of the anus.

*Cholera Morbus.*–Of the six cases registered as cholera morbus, or serous diarrhœa with vomiting, none proved fatal, though one was in a state of collapse when brought in, and the re-action was followed by a most severe and obstinate dysentery. Two of the cases bore all the characteristic features of *epidemic* cholera, including the cramps, rice-water discharges, &c.

*Pneumonia.*—Eleven cases were registered under this head, three of which proved fatal. Of the latter, one was admitted Jan. 28th, with extensive pneumonic inflammation, complicated with enlargement of the liver and spleen, and œdema of the extremities. His pulse was feeble and respiration very difficult. He died the following day, aged 20 years. This case was evidently the sequel of severe periodical fever during the autumn previous.

The second fatal case was that of a German, aged 60 years, admitted April 5th, and died on the 7th of the same month. He was too much exhausted to give any reliable account of the length of time he had been sick, or the manner of its commencement. The third fatal case was also a German, aged 35 years. He was admitted to the hospital on the 8th and died on the 11th of June.

The only entry in the register on the day of admission is as follows, viz.: "Pneumonia with extensive hepatization of both lungs; prognosis exceedingly unfavorable."

The remaining eight cases presented the disease in almost every stage of progress, although but one was admitted before the fourth day after the commencement of the attack. Most of them were from the ranks of the poor, and the general febrile symptoms decidedly typhoid in their character. It is necessary to bear these facts in mind, as they have an important bearing on the treatment. In none of the cases was general bleeding deemed advisable, and so strong was the tendency to prostration with that intestinal irritation which generally accompanies typhoid fever, that the tartrate of antimony and potassa could be given with safety only in combination with opium. The following case will serve as a fair illustration of the whole:

Mr. J. M., native of Ireland, aged 22 years, was admitted into the hospital July 18th, 1853. He says he has been sick four or five days, and has taken some physic. At present his face is flushed, eyes slightly injected and heavy, breathing short and labored, tongue covered with a thin whitish yellow fur and moist, frequent cough with the expectoration of muco-purulent matter intermixed with blood, pulse 95 per minute and soft, skin dry and little above the natural temperature; pain in the head and right side, greatly increased by coughing; urine scanty and high colored, and bowels moved once or twice during the last 24 hours. On percussion the infra-clavicular and mammary regions on the right side were found decidedly duller than natural. There was also in some parts of the same regions, coarse mucous ronchus with increased vibration of voice, and in other parts plain crepitant rale.

The patient was directed to take the following powder every four hours, viz.: ℞ Pulv. Doveri. 8 grs.
Sub. Murias Hydrarg. 1 gr.
Tart. Ant. et Pot. 1–6 gr. mixed.

And between each dose of the powders one teaspoonful of the following mixture, viz.:

℞ Comp. Honey of Squills and Senega, ℥j.

Tinct. Opii. at Camp., ℥j. mixed.

July 19th. Cough less frequent and expectoration more copious, otherwise symptoms nearly the same as yesterday. Continue same treatment with the addition of a large blister over the upper part of the chest. Allow a small quantity of beef-tea for nourishment.

July 20th. Respiration more full and less frequent; expectoration copious and muco-purulent, but less mixed with blood; pulse 90 per minute and feeble; skin moist and cool; no head-ache but still some soreness in the chest. Blister had drawn well. Directed the powders of opium, antimony, &c., to be continued, and a powder composed of sulphate of quinine 5 grs., and chloride of sodium 15 grs. each night and morning. Omit the expectorant mixture.

July 21st. Bowels moved rather too freely this morning. Gums show slight indications of mercurial action; feels much prostrated, but all other symptoms improved. Directed all previous medicine to be omitted, and gave chloride of sodium 10 grs. with pulv. opium 1 gr. every four hours, with a teaspoonful of the emulsion of oil of turpentine and laudanum between each dose of the powders.

22d. Patient improving. Bowels less freely moved. Directed the same powders to be continued, and the former expectorant mixture in the place of the emulsion.

23d. Still improving; continue same treatment.

24th. Respiration quite free; dulness on percussion nearly disappeared; pulse 80 per minute: expectoration less in quantity, thick and yellow, and without blood. Has some appetite. Directed the powders to be continued three times a day, alternated with the expectorant mixture.

27th. The patient has continued to improve since the 24th, and was discharged to-day feeling quite well.

The average of the treatment in the whole eight cases in which recovery took place, was little more than ten days; the shortest being six and the longest fourteen days.

*Pleurisy.*—Of the four cases of pleurisy, one was connected with tubercular deposits in the lungs, one became complicated with a post-pharyngial abscess, and the remaining two were simple cases attended with moderate effusion. The second case named presented some points of sufficient interest to justify a brief history.

John Ryan, aged about 35 years, a native of Ireland, was admitted into the hospital March 8th, 1853. He stated that two weeks previous he had been attacked with pain in his right side, shortness of breath, dry cough, and high fever. At present his respiration is short and hurried; pulse 100 per minute, firm, but not full; inability to lie on the left side without a sense of suffocation; short dry cough; sharp pain in the right side of the chest on coughing or attempting to take a full inspiration; urine scanty and high colored; some thirst; and tongue covered with a thin whitish fur. On inspection of the chest, the right side around the mammary region was found enlarged and the intercostal spaces fuller than the opposite side. The same region was also dull on percussion, and over much of it an entire absence of the respiratory murmur, with bronchial respiration under the clavicle.

These signs and symptoms showed clearly the existence of pleuritic inflammation, with effusion in the right side of the chest. He was directed a powder composed of sub. murias. hydrarg. 1 grain, pulv. digitalis, 1 gr., and nit. potassa 5 grs. every four hours, with pulv. doveri and nit. pot., each 5 grs., at bed-time, and a blister to the affected side. He continued his treatment four days with a slow but decided improvement in all his symptoms. On the 13th, his gums and salivary glands beginning to show signs of mercurial action, the calomel was omitted from the powders and pulv. doveri 5 grains, substituted in its place, and an additional blister applied to the chest. He continued this until the morning of the 16th, when the symptoms both of inflammation and effusion into the chest were much diminished. His respiration was much more full, and the dullness over the right side much diminished. His gums being moderately sore with an increased flow of saliva from the mercurial previously taken, and the bowels slightly costive, he was directed 5 grs. each of pulv. rhei, and bi-carb. soda, and the same to be repeated at night if the first did not operate. Also a wash of chloride of zinc, one grain to one ounce of water for his mouth. All other medicine was discontinued.

The next morning, (the 17th,) his bowels had moved too many times, exhibiting a dysenteric tendency, and he complained of great pain on attempting to swallow, and a feeling like that produced by a foreign body in the throat. Inspection of the throat revealed nothing but slight enlargement of the tonsils and a blush of redness over the fauces and pharynx. He was directed to take a teaspoonful of the emulsion of oil turpentine and laudanum every four hours to control the bowels, and to continue the same wash for his mouth and throat.

18th. Found the bowels still loose, and the difficulty of swallowing greatly increased with equal difficulty of breathing; the latter being of that peculiar character indicating direct obstruction of the larynx. There was also complete loss of voice. His pulse was 100 per minute, small, sharp, and quick; also, general restlesness arising from the sense of suffocation.

A strong solution of nitrate of silver was applied with the sponge to the larynx and fauces, and directed a blister to be applied to the side of the neck. Owing to the great difficulty of deglutition he was directed only the following powder at night, viz.: ℞ Pulv. Doveri 8 grs., and Pulv. Antimonialis 2 grs.

19th. Breathing extremely difficult, even threatening suffocation; aphonia complete; the whole trachea swollen and tender to the touch; pulse small and quick; skin bathed with perspiration; swallowing almost impossible; countenance appears anxious and bloated. Ordered an enema of castor oil to open the bowels, and fomentations with warm infusion of aconite leaves to the neck.

March 20th. Symptoms nearly the same. More swelling of the larynx and trachea externally. The inspirations produce a sound like a current of air through a contracted and dry tube. Continue same treatment.

21st. Symptoms but little changed; the sense of suffocation is somewhat less; and the swelling more prominent externally on the left side of the trachea. Directed him to take a powder composed of Pulv. Doveri 6 grs.
Pulv. Antimonialis 1 gr.
Blue Mass 1 gr., mixed, every four hours if he could swallow it, and continued the fomentations externally.

22d. No improvement. Patient had chills this morning; pulse small and quick; breathing and swallowing very difficult and painful. The swelling is still more prominent on the left side of the neck, and it is evident that suppuration has taken place, though no distinct fluctuation could be detected; and it was too low down to be reached through the mouth. Directed a flax-seed meal poultice in place of the fomentations, and continue the powders.

23d. No change, except a very obscure sense of fluctuation in the swelling, and much general prostration. Made a deep incision on the left side of the trachea a little below the lower margin of the thyroid cartilage, and gave exit to a large quantity of thick well formed pus, which afforded much relief to the breathing. The poultice was re-applied and the patient left at rest.

24th. Respiration much easier, pulse more full, skin dry and warm, but swallowing very difficult, and still entire suppression of voice, with a troublesome inclination to cough. There is still much swelling and hardness around the larynx and trachea, and considerable discharge of pus. A probe can be passed through the opening deep behind the trachea, revealing an abscess of considerable extent. To counteract a slight febrile action and allay the cough, he was directed to take a tablespoonful of the following solution with half a teaspoonful of tinct. of opium and camphor, every four hours, viz.: ℞ Tart. Ant. 1 gr.
Gum. Arabac. ℥j·
Water, half a pint, mixed.

From this time on the patient gradually improved. The abscess continued to discharge eight or ten days, and another, of small extent, however, formed directly on the front part of the trachea just below the thyroid cartilage.

On the first of April the cough had subsided; the respiration and deglutition had become comparatively easy; and the voice much more distinct. The inflammatory action having subsided, and there being much debility with still some discharge from the abscesses, he was directed to take three times a day the following prescription, viz.: ℞ Ext. Sanguinis, 10 grs.
Pulv. Rhei. 3 grs.
Bi-Carb. Soda. 3 grs., mixed.

He was also allowed animal broth for nourishment. This treatment was continued until April 11th, with no other troublesome symptom than a severe spasmodic action in the sterno-cleido-mastoid and some of the deeper muscles of the left side of the neck. This began about the time the abscess ceased to discharge, and continued very painful at times for two or three days. It was apparently relieved by the external application of the following mixture, viz.: Sat. Tinct. Anconite Root, ℥j.
Camphorated Oil, ℥j.

On the 11th, there being still much debility, with pretty copious night sweats, he was directed to discontinue the former powders and take the following in their place, viz.:

Sulph. Quinine, 2 grs.
Chloride Sodium, 10 grs.
Prussiate of Iron, 2 grs., mixed ft. 1 powder.

He continued to improve rather slowly, and was discharged quite well on the 23d of the month, the original pleuritic affection being apparently well.

*Remarks.*—Abscesses posterior to the trachea or œsophagus are comparatively rare, and their diagnosis in the early stage not always clear and satisfactory. Another item of interest, was the fact that the abscess commenced and progressed to completion at a time when the patient was sufficiently under the influence of mercury for the pleuritic disease, to cause a moderate degree of salivation. It may be well also to drop a remark here in reference to the external application of anconite to painful phlegmonous swellings.

During the last two or three years I have been in the habit of using the infusion of aconite leaves, to allay the pain and cause the resolution of carbuncles and other phlegmonous swellings tending to suppuration; and where it is applied early and constantly, it is often very effectual. I generally direct half an ounce of the leaves to one pint of boiling water, and keep the part constantly covered with a cloth dipped in the infusion.

I have also frequently applied the same infusion to joints very painful from rheumatic inflammation, and with much temporary relief.

